# The current situation regarding the availability and accessibility of anticancer drugs for breast cancer in the Peruvian public health systems

**DOI:** 10.3332/ecancer.2021.1224

**Published:** 2021-04-27

**Authors:** Guillermo Valencia-Mesías, Patricia Rioja-Viera, Zaida Morante-Cruz, Yura Toledo-Morote, Silvia Neciosup-Delgado, Henry Gómez-Moreno

**Affiliations:** 1 Medical Oncology Department, National Institute of Neoplastic Diseases, Lima 15036, Peru; 2 Functional Insurance Unit, National Institute of Neoplastic Diseases, Lima 15036, Peru

**Keywords:** breast cancer, Peru, high-cost drugs, accessibility, INEN

## Abstract

The availability of effective, accessible, safe and high-quality anticancer drugs for the medical treatment of cancer is fundamental in ensuring optimal healthcare in the public health system in Peru. The main objective is to assess the current situation regarding anticancer drugs (termed ‘high-cost’) for breast cancer, as well as an analysis of the possible factors which negatively impact access to the Peruvian public health systems.There are similarities in the availability of anticancer drugs, since most treatments are covered by the Peruvian Ministry of Health. EsSalud offers an extra monoclonal antibody (pertuzumab) in the metastatic treatment stage. Meanwhile, the National Institute of Neoplastic Diseases (INEN), mainly for metastatic disease, has relied on more tested treatments over the last year. An agreement has been reached with the armed forces, which will enable patients to receive oncology care at the INEN and, thereby, benefit from the use of high-cost drugs.

## Introduction

*The current situation on the availability and accessibility of anticancer medications for breast cancer in Peru*

Cancer treatment is very expensive. Anticancer medications for breast cancer, and biological treatments in particular (monoclonal antibodies), are considered to be high-cost. These treatments are too expensive to be paid for as ‘out-of-pocket’ expenses. Therefore, the need for public insurance is all but mandatory for patients with human epidermal growth factor receptor 2 (HER2) (+) breast cancer, who will receive monoclonal antibodies. One dose of trastuzumab (by injection), used for HER2 (+) breast cancer, costs 2,861.25 sols (S/.) (by injection) and S/. 4,722.11 (subcutaneous (SC)); a neo/adjuvant treatment (18 cycles) by injection can cost S/. 51,502.25 (55.3 minimum wages).^1^ Likewise, one dose at the start of treatment costs 3–5 months’ work for a person earning the minimum wage [1].

Another one of the main reasons that contribute to the interruption of oncology treatment is geography. It has been estimated that 51% of patients come from the provinces. It can be devastating for families who live outside of the large cities, not to have a specialised cancer health centre close to home. If the patient has to be moved from one place to another, resources provided by the public health system are minimal, and ‘out-of-pocket expenses’ during the move (tickets, food, accommodation) have a significant impact on the family's finances. It is a constant drama to imagine the situation of the patients who have to travel long distances to get a definitive diagnosis and, eventually, the optimal treatment for the cancer from which they suffer. The people living in poverty or extreme poverty are those who suffer the greatest impact from this situation and are those who stop treatment. When they go to the specialised public cancer centres (located in the main capitals), the patients don't actually have the financial means to pay for their tickets and their stay, this is also associated with other family, idiosyncratic, psychological and cultural factors [1, 2].

A Peruvian study noted that the highest percentage of cancer diagnoses is made when the disease is at an advanced stage (up to 75% of diagnosed cases) [2]. The Metropolitan Lima Cancer Registry (2010–2012) showed that the three most common types of cancer in women were: breast, cervix and stomach [3]. Another Peruvian study, which assessed the existing barriers in Latin America (LATAM) for the management of locally advanced breast cancer (ABC), noted four key pillars: 1. a high burden of disease (late diagnosis in advanced stages), 2. inadequate access to health services, 3. insufficient access to specialised cancer centres and 4. Insufficient research into breast cancer in LATAM [4]. Likewise, a study carried out in LATAM that assessed access to high-cost drugs for ABC, particularly trastuzumab, showed that while more than 60% of patients in the United States are diagnosed in the early stages of the disease, the data for Brazil and Mexico indicates that more than 60%–70% of cases are diagnosed in more advanced stages, require more complex management and have lower chances of recovery [5].

The Peruvian health system is fragmented and this characteristic is a cause of the underperformance of the healthcare systems and services. There are three different types of public insurance (named IAFAS – Administrative Institutions of Health Insurance Funds) that enable the care of patients in particular institutes (named IPRESS – Institutions Providing Public Healthcare Services): Peruvian Ministry of Health (MINSA), EsSalud and the Armed Forces. Patients can only be treated by their respective insurance company [6]. Comprehensive Health Insurance (Seguro Integral de Salud (SIS)) and the Intangible Solidarity Health Fund (Fondo Intangible Solidario de Salud (FISSAL)) are mainly intended for people living in poverty and extreme poverty; they depend on MINSA. EsSalud, created exclusively for the care of the working class, depends on the Peruvian Ministry of Labour (MINTRA). The Health Insurance Fund of the National Police of Peru (SALUDPOL), which is a type of insurance for police officers, depends on the Ministry of the Interior. The Health Funds for the Armed Forces Military Personnel which depend on the Ministry of Defence are: IAFAS of the Peruvian Army (FOSPEME), IAFAS of the Peruvian Navy and IAFAS of the Peruvian Air Force. Each of them has financial, budgetary and accounting independence. As the governing body, MINSA regulates the health policies for the Peruvian people and may oversee other healthcare systems. The National Institute of Neoplastic Diseases (INEN) also has the role of regulating cancer care in Peru [6].

Although each insurance is independent, each one has specific healthcare policies and plans for its cancer patients. It would fall to FISSAL to contribute additional funds for the provision of treatment, given that FISSAL funds should be used for all high-cost illnesses, rare and orphan diseases as well as high-cost procedures. The coverage includes the financing of PNUME (a single national list of essential medications) and non-PNUME medications in accordance with the existing regulations (RM 540-2011/MINSA, RM 1361-2018/MINSA and RM 862-2019/MINSA) [1–7].

Peru has made progress in the fight against cancer. In 2006, MINSA and contributing institutes developed multi-sectoral cancer control strategies, focused on prevention, education, early detection and the expansion of services for numerous cancers. For breast cancer, the plan involved individual and group advice on breast cancer prevention (women aged between 18 and 64) as well as the promotion of annual mammograms (group aged between 40 and 65 years). In 2009, SIS began to provide oncology care. In 2011, a cancer prevention and control programme was created and implemented with an initial budget of S/. 30.5 million. In 2012, ‘Plan Esperanza’ (Hope Plan) was created, with this programme receiving a budget of S/. 125.8 million, spread across 25 regions. The plan is intended to cover people living in poverty and extreme poverty who are members of SIS, which is why it became the cancer insurance with the highest number of members. This programme covers seven types of cancer (cervical, breast, stomach, colon, prostate, lymphomas and leukaemias). More than 16 million people fighting cancer were covered by SIS, making this being classified as one of the most expensive diseases in its treatment and whose early detection requires the best conditions and resources available [2–8].

With regard to oncology care in Peru, although MINSA has four specialised oncology institutes (INEN, Regional Institute of Neoplastic Diseases (IREN) North, IREN Central and IREN South), some healthcare establishments in various provinces ‘act’ as cancer treatment centres for others that don't have medical oncology services in the same region. For example, Piura (located in northern Peru) receives patients from Tumbes, since this department does not have oncologists nor chemotherapy in its public MINSA hospitals. A similar situation happens in Ica (located in the south), which treats patients that live close to this region (mainly Ayacucho and Huancavelica). Table 1 outlines the medical oncology services available nationwide, with the presence of at least one oncologist in said region; the total number of active oncologists (319 affiliated to Peruvian Society of Medical Oncology (SPOM)) and calculates the number of oncologists/100,000 residents (and a comparison with the United States and Spain – Spanish Society of Medical Oncology (SEOM)).

## Methodology

For the preparation of this manuscript, telephone and email interviews were conducted with representatives of the main public health systems involved in cancer care, in the capital (Lima) and the provinces. These were divided according to type of public insurance (SIS, EsSalud, Armed Forces). National opinion leaders in breast cancer management were also contacted. Moreover, information was collected from SPOM regarding the availability of oncologists and medical oncology services on a national level, including calculations on the number of oncologists per 100,000 residents and their comparison with other countries (Table 1). Information was also collected about access, types of medication available for breast cancer and their costs from the Functional Insurance Unit and the Pharmacy of INEN.

## Results

### ‘High-cost’ anticancer medications

In 2017, the European Society of Medical Oncology (ESMO) presented a report on costs and accessibility to cancer medications based on the World Health Organisation's (WHO) Model List of Essential Medicines. Peru has access to more than 90% (22/24) of the list of anticancer medicines outlined in said report [9].

In Peru, a medication is considered ‘high cost’ when it exceeds two tax units (2 (Peruvian) Fiscal Tax Units (UITs)) (In 2021, 1 UIT is equivalent to S/. 4,400) [10]. This definition corresponds to extraordinary coverage. However, in Peru, there is no regulation which defines high-cost medicines and even the procedure for funding high-cost health technologies has yet to be defined [11]. Cytotoxic treatments (chemotherapy) don't exceed 2 UITs, while the cost of monoclonal antibodies (like intravenous trastuzumab) is equivalent to 65% of 1 UIT. In the last 5 years, there has been a declining trend in the price of anticancer medications for breast cancer, especially trastuzumab.

### Availability of high-cost medications for breast cancer depends on the type of insurance

In Peru, the funding of every type of insurance is different. In the case of the specialised oncology institutes (INEN, IREN North, Central and South), MINSA provides the funding through FISSAL. Approval of new high-cost medications not already considered by PNUME requires two conditions (according to RM 862-2019/MINSA). Level III-2 healthcare facilities may acquire and/or use medications, not included in the PNUME, previously evaluated and approved by their Pharmacotherapeutic Committee (responsible for the evaluation of medication and medical devices). This medication must be contained in a clinical practice guideline (CPG) or policy document framed by MINSA's Policy Documentation Development Regulations [12].

In July 2019, INEN presented technical support for the approval of new medications not included in the PNUME. As a result, more breast cancer treatments were approved, primarily for the metastatic stage: lapatinib (in combination with capecitabine in patients with HER2 (+) metastatic breast cancer who have previously received anthracycline, a taxane and trastuzumab); fulvestrant (a monotherapy for patients with luminal (hormone receptor positive/HER2 (−)) breast cancer not previously exposed to endocrine therapy or who have previously been exposed to endocrine therapy); ixabepilone (a monotherapy for patients with triple negative breast cancer who have previously received anthracycline, a taxane and capecitabine); liposomal doxorubicin (metastatic breast cancer that has progressed to lines of treatment) and SC trastuzumab (indicated only as an adjuvant therapy). This has provided incredible access and treatment opportunities for selected patients. Regarding endocrine therapy, fulvestrant has a higher cost compared to other available endocrine therapies. The list of medicines available at INEN and their costs are shown in Tables 2 and 3; their conditions of use are detailed in Table 4. Additionally, INEN is currently updating their CPGs and institutional policy documents (for example: technical documents (TDs)) that support the use of these medications, including breast cancer. INEN has a breast cancer CPG (2013) [13] and recently the ‘Multidisciplinary management of neo/adjuvant HER2 (+) and triple negative breast cancer’ (2019) [14] TD has been approved. Furthermore, a TD for the oncological treatment of metastatic breast cancer is in the process of being approved.

The regional specialised institutes (IREN North, Central and South) only have standard cytotoxic therapy (without liposomal doxorubicin or ixabepilone), intravenous trastuzumab and endocrine therapy based on tamoxifen and anastrozole (without exemestane or fulvestrant, except IREN North which does have exemestane) for the treatment of breast cancer.

With regard to EsSalud, funding depends on MINTRA. The approval of new medications under this type of insurance depends on a committee from an Evaluation of Healthcare Technology (named IETSI – Institute for the Evaluation of Health Technologies and Health Research), which develops CPGs and generates recommendations on the usage of new and existing medicines within EsSalud. For HER2 (+) breast cancer, this system has the approval of pertuzumab (monoclonal antibody) in the metastatic stage, with an approximate cost per unit 3.6 times more expensive than that of intravenous trastuzumab (S/. 8,745.24 and S/. 2,400.00, respectively)**.** Furthermore, intravenous trastuzumab is approved for use during the neo/adjuvant and metastatic phases with EsSalud. SC administration is not available.

In the case of the police and the armed forces, financing is through an internal health insurance fund (named SALUDPOL and FOSPEME for the police and military, respectively). They have access to breast cancer medications in a similar way to the regional MINSA institutes. In November 2019, an agreement was signed between INEN and SALUDPOL guaranteeing access to specialised oncology services. SALUDPOL has signed an agreement to allow access for more than 450,000 police officers and their families to cancer care at INEN, due to the negative impact treatment costs have on their health system. Consequently, the patients and families affiliated with SALUDPOL will benefit from high-cost medications [15].

## Discussion

*Approval and cost of trastuzumab for breast cancer according to stage (adjuvant, neoadjuvant, metastatic)*

Trastuzumab, a recombinant humanised monoclonal antibody, is an effective form of treatment used in HER2 (+) breast cancer treatment, as it reduces the risk of local and distant recurrence, as well as being associated with increased survival. This medication has a relatively high cost compared to other breast cancer drugs (chemotherapy, endocrine therapy), especially in developing countries. Trastuzumab was included in the WHO's Model List of Essential Medicines for the treatment of early and HER2 (+) metastatic breast cancer [16].

The evidence and pharmacoeconomic evaluation in developed countries show that it is a cost-effective medicine. However, despite being considered an essential medicine by WHO and having a proven clinical benefit in HER2 (+) breast cancer, some studies have not found trastuzumab to be cost effective in various LATAM countries. A study of the cost effectiveness of trastuzumab in six LATAM countries (Argentina, Bolivia, Brazil, Chile, Peru and Uruguay) showed that it was not cost effective (using WHO's definition of cost effectiveness of less than 3 times the gross domestic product per capita per quality adjusted life year) [17]. Another similar study of cost effectiveness in Colombia indicated that trastuzumab was not cost effective [18]. A study of cancer control interventions in Peru (2013) showed that the clinical examination and fine needle biopsy of breasts in non-urban areas (for patients between 40 and 69 years of age) combined with a clinical breast examination and mammogram in urban areas (for patients between aged between 40 and 69 years) could result in cost effective and feasible options; while therapy with trastuzumab and the management of metastatic disease are not economically attractive options [19].

In August 2017, comprehensive cancer care and improving access to oncology services were declared of national interest in Peru. In December 2018, PNUME for oncology was approved for the health sector [20]. Trastuzumab was indicated only as an adjuvant therapy in patients with HER2 (+) breast cancer. In June 2019, an amendment was made, approving the use of trastuzumab as a neoadjuvant therapy [21]. In July 2019, trastuzumab was approved for use as a first line treatment in metastatic breast cancer at INEN. In December 2019, the suggested use for trastuzumab was extended as far as the third line treatment (as a first line of treatment in combination with taxanes; a second line of treatment combined with taxanes in patients who received anthracyclines; a third line of treatment in combination with capecitabine or vinorelbine in patients who received anthracyclines and taxanes).

The cost of trastuzumab used in neo/adjuvant regimes as well as in metastatic disease has reduced in price by approximately half (Table 5) due to the use of biosimilar drugs at INEN. The first batch of one biosimilar drug arrived by direct purchase by National Centre for the Strategic Supply of Health Resources (CENARES) (responsible for the adequate supply of resources, ensuring their availability and the best market conditions in public establishments) in October 2019, it began to be used in February 2020. A study carried out in LATAM previously mentioned that the use of biosimilars has the potential to improve the access to breast cancer therapies, providing a reliable alternative when making treatment decisions. In some countries such as India and Korea, a trastuzumab biosimilar has been approved for the metastatic stage [5]. The latest international consensus recently published by ESMO for ABC strongly recommends the use of biosimilars for both biological treatment (for example: trastuzumab), as well as for supportive care (for example: colony stimulating factors), after passing the rigorous validation procedures required by the high-level health surveillance bodies (such as the Food and Drug Administration or European Medicines Agency, for example) [22].

The time taken to approve a ‘high-cost’ medicine such as trastuzumab at INEN (from recommendation by a medical board to dispensing the drug) used to be approximately 45 days. Currently, the process takes between 48 hours and 1 week.

## Conclusions

In Peru, cancer treatment is very costly. Multiple factors contribute to the poor access to high-cost medicines, the principle causes being: fragmentation of the Peruvian healthcare system, costs, geography, out-of-pocket expenses during transfer to oncology centres, idiosyncrasies, economic conditions, centralisation of cancer care, among others. In the case of MINSA's specialised oncology institutes, INEN has had greatest access to new high-cost medicines approved in 2019, primarily for the metastatic stage (liposomal doxorubicin, fulvestrant, ixabepilone, lapatinib) and approval for the adjuvant therapy (SC trastuzumab). The other regional MINSA institutes only have cytotoxic therapies, intravenous trastuzumab and endocrine therapies based on tamoxifen and anastrozole. The approval time for high-costs drugs has currently reduced drastically (INEN), providing rapid access to these medicines. Regarding EsSalud, this system has approved pertuzumab for the metastatic stage, this being the main difference from MINSA. In the case of the police and armed forces, they have approved medications similar to those of MINSA's oncology centres (except INEN). Recently an agreement between SALUDPOL and INEN has been signed, to provide oncology care to the police and their families, offering a great opportunity for better access to high-cost medications that are not currently approved in said system.

A downward price trend in high-cost breast cancer medications has been observed both in MINSA and EsSalud, particularly with trastuzumab (which is approximately at half of its previous price), which generates greater access to this therapy. The main factor is the emergence and use of a trastuzumab biosimilar provided by CENARES. Regarding chemotherapies, they have maintained their usual price for the last 5 years. In the case of new endocrine therapies, INEN currently relies on exemestane and fulvestrant, the latter being the most expensive medication compared to the rest of endocrine therapies. Even if a reduction in the price of breast cancer treatment has been demonstrated, it emphasises the need to take into account cost-effectiveness studies carried out in LATAM. This is due to the fact that previous ones showed that trastuzumab was not cost effective, even when compared to other treatments. Peru has gone to great lengths in the fight against cancer over the last 10 years. However, despite all this effort there is a lot left to do and the coverage of breast cancer continues to be a great challenge for Peruvian public health systems. Transparent evaluation and medication approval processes will highlight many of the local problems which are not displayed during the administrative process.

## List of abbreviations

ABC, Advanced breast cancer; CENARES, National Centre for the Strategic Supply of Health Resources; TD, Technical document; ESMO, European Society of Medical Oncology; EsSalud, A Peruvian Social Health Insurance Company; FISSAL, Intangible Solidarity Health Fund; FOSPEME, Healthcare Fund for Army Military Personnel; CPGs, Clinical practice guidelines; HER2, Human epidermal growth factor receptor 2; IAFAS, Administrative Institutions of Health Insurance Funds; INEN, National Institute of Neoplastic Diseases; IREN, Regional Institute of Neoplastic Diseases; LATAM, Latin America; MINSA, Peruvian Ministry of Health; MINTRA, Peruvian Ministry of Labour; PNUME, Single National List of Essential Medicines; SALUDPOL, Health Insurance Fund of the National Police of Peru; SEOM, Spanish Society of Medical Oncology; SIS, Comprehensive Health Insurance; SPOM, Peruvian Society of Medical Oncology; UIT, (Peruvian) Fiscal Tax Unit; WHO, World Health Organization

## Conflicts of interest

None of the authors have a conflict of interest.

## Funding statement

The authors have no funding to declare.

## Authors' contributions

GVM and PRV conceptualised, designed the manuscript, carried out data collection and redacted the manuscript. ZMC, YTM, SND, HGM carried out the analysis, critical review of the manuscript, as well as data interpretation and completed the final edition. All the authors approved the final version of the article.

## Figures and Tables

**Table 1.** Medical oncology care situation in Peru-2020.

**Number of active oncologists [1]:** Total number of oncologists affiliated with the SPOM (including paediatric oncologists): 319 Distribution by department (most common): Lima: 63% Arequipa: 10.03% Lambayeque: 5.64% La Libertad: 5.32%			
**Availability of medical oncology services and the presence of at least one oncologist in public healthcare systems at a national level – Peru [1]**			
**Macro regions**		**Medical oncology service**	**Oncologist**
**North**			
Ancash			
La Libertad			
Piura			
Cajamarca			
Lambayeque			
Tumbes			
**South**			
Arequipa			
Apurimac			
Cusco			
Moquegua			
Puno			
Tacna			
**Central**			
Ica			
Junin			
Ayacucho			
Pasco			
Huancavelica			
Huánuco			
**Selva**			
Madre de Dios			
Loreto			
San Martín			
Amazonas			
Ucayali			
**Lima y Callao**			
Lima Metropolitan Area			
Lima region			
Callao			
**Key:**			
Yes			
No/data unavailable			
**Number of medical oncologists per 100,000 inhabitants (2020):** Peruvian population: approximately 32,000,000 people Number of oncologists (affiliated with SPOM): 319 Number of oncologists/100,000 inhabitants: **0.99** In the USA: there are 3–4 oncologists/100,000 inhabitants (a statistic considered low for their population) [2]. In Spain: 1–2 oncologists/100,000 inhabitants (SEOM considers an acceptable standard of care to be 1–2 oncologists per 100,000 inhabitants or 15–20 specialists/million inhabitants) [3, 4]. A Peruvian report (February 2019) showed that they have two oncologists (including surgical oncologists, oncologists, paediatric oncologists, clinical oncologists, pathological oncologists)/100,000 inhabitants [2]. Using this data, 0.93 oncologists/100,000 inhabitants are produced.			

Sources: Peruvian Society of Medical Oncology (SPOM). 2020; Barja L. Día mundial contra el cáncer: hay sólo dos oncólogos por cada 100 peruanos. Peruvian Radio Programmes (RPP) news report. 2019; Cubedo R. Varias cifras sobre la oncología española. elmundoes. Health. 2006; Casas Fernández de Tejerina A. Recomendaciones de recursos en el bloque asistencial. Primer libro blanco de la Oncología Médica en España. Spanish Medical Oncology Society (SEOM). 2006

**Table 2.** Accessibility to high-cost anticancer medications in Peru.

Anticancer medication	INEN (Lima) – MINSA	IREN North (Trujillo) – MINSA	IREN Central (Junín) – MINSA	IREN South (Arequipa) – MINSA	EsSalud
IV Trastuzumab					
SC Trastuzumab					
Pertuzumab 420 mg					
Lapatinib 250 mg					
Doxorubicin 50 mg					
Cyclophosphamide 1,000 mg					
Paclitaxel 100 mg					
Paclitaxel 30 mg					
Docetaxel 80 mg					
Docetaxel 20 mg					
Carboplatin 450 mg					
Carboplatin 150 mg					
Capecitabine 500 mg					
Liposomal doxorubicin 2 mg/mL/10 mL					
Ixabepilone					
Tamoxifen 20 mg					
Anastrozole 1 mg					
Exemestane 25 mg					
Fulvestrant 250 mg					

The nomenclature of IV trastuzumab used at INEN is 21 mg/mL. It is available in two packages (vials): Herceptin (440 mg/5 mL) and a biosimilar (Bisintex 420 mg/20 mL)

The SC trastuzumab vial is 120 mg/5 mL

Sources: Cherny NI, Sullivan R, Torode J, Saar M, et al. ESMO International Consortium Study on the availability, out-of-pocket cost and accessibility of anticancer medicines in countries outside of Europe; Pharmacy Department. National Institute of Neoplastic Diseases (INEN); Pharmacy Department. Ramiro Prialé Prialé National Hospital. EsSalud

**Table 3.** Price list of breast cancer medications – INEN.

Anticancer medications	Unit cost 2016	Unit cost 2017	Unit cost 2018	Unit cost 2019	Unit cost 2020
**Monoclonal antibodies**					
IV Trastuzumab	S/. 6,566.10	S/. 6,500.43	S/. 6,500.43	S/. 5,674.89	S/. 2,861.25
SC Trastuzumab	-	-	-	S/. 4,722.11	S/. 4,760.20
**Targeted therapy**					
Lapatinib 250 mg	-	-	-	-	S/. 43.53
**Cytotoxic therapy (chemotherapy)**					
Doxorubicin 50 mg	S/. 20.00	S/. 20.00	S/. 19.00	S/. 19.00	S/. 19.00
Cyclophosphamide 1,000 mg	S/. 225.30	S/. 225.30	S/. 81.25	S/. 61.25	S/. 26.00
Paclitaxel 100 mg	S/. 30.00	S/. 21.00	S/. 22.00	S/. 23.12	S/. 17.00
Paclitaxel 30 mg	S/ 9.30	S/. 9.30	S/. 9.30	S/. 14.46	S/. 17.00
Docetaxel 80 mg	S/. 50.00	S/. 42.00	S/. 42.00	S/. 81.25	S/. 26.24
Docetaxel 20 mg	S/. 31.25	S/. 37.50	S/. 28.12	S/. 27.00	S/. 18.04
Carboplatin 450 mg	S/. 64.40	S/. 84.40	S/. 64.40	S/. 83.75	S/. 78.00
Carboplatin 150 mg	S/. 34.00	S/. 23.00	S/. 23.00	S/.31.23	S/. 30.00
Capecitabine 500 mg	-	-	-	-	S/. 0.88
Liposomal doxorubicin 2 mg/mL/10 mL	-	-	-	-	S/. 187.50
Ixabepilone	-	-	-	-	S/. 1,720.68
**Endocrine therapy**					
Tamoxifen 20 mg	-	-	-	-	S/. 0.16
Anastrozole 1 mg	-	-	-	-	S/. 0.13
Exemestane 25 mg	-	-	-	-	S/. 3.23
Fulvestrant 250 mg	-	-	-	-	S/. 1,178.00

The nomenclature of IV trastuzumab used at INEN is 21 mg/mL. It is available in two packages (vials): Herceptin (440 mg/5 mL) and a biosimilar (Bisintex 420 mg/20 mL)

The SC trastuzumab vial is 120 mg/5 mL

Sources: Pharmacy Department. National Institute of Neoplastic Diseases (INEN); Functional Insurance Unit. National Institute of Neoplastic Diseases (INEN)

**Table 4.** Terms of use for non-PNUME breast cancer medications approved by INEN-2020.

No	Assessed medication			Type of cancer	Terms of use	CPGs
	International nonproprietary name (INN)	Concentration	Pharmaceutical form			
1	Exemestane	25 mg	Tablet	Breast cancer	Treatment of metastatic breast cancer in progression to anastrozole or in those who are intolerant of this.	Yes
2	Liposomal doxorubicin	2 mg/mL/10 mL	Injectable	Breast cancer	Metastatic breast cancer with progression to two or more lines of treatment.	Yes
3	Fulvestrant	250 mg/5 mL	Injectable	Breast cancer	As a monotherapy for the treatment of hormone sensitive metastatic or recurring breast cancer in post-menopausal women who have not received endocrine therapy, or who have previously progressed to endocrine therapy.	Yes
4	Ixabepilone	45 mg	Ampoule	Breast cancer	Monotherapy in patients with locally advanced or metastatic breast cancer who have previously received therapy, including an anthracycline, a taxane and capecitabine.	Yes
5	Trastuzumab	440 mg/5 mL	Injectable	Breast cancer	First line treatment in combination with taxanes in patients with metastatic, overexpressed or amplified HER2 breast cancer.	Yes
					Second line treatment in association with taxanes in patients who received anthracyclines as first line treatment.	
					Third line treatment (in association with anti-oestrogen therapy or with capecitabine or vinorelbine) in patients who have previously received anthracyclines and taxanes.	
6	Lapatinib	250 mg	Tablet	Breast cancer	In combination with capecitabine for the treatment of patients with HER2 metastatic breast cancer who have previously received therapy, including an anthracycline, a taxane and trastuzumab.	Yes
7	Ixabepilone	15 mg	Ampoule	Breast cancer	Monotherapy in patients with locally advanced or metastatic breast cancer who have previously received therapy, including an anthracycline, a taxane and capecitabine.	Yes

Source: Pharmacotherapeutic Committee – National Institute of Neoplastic Diseases (INEN). 2020

**Table 5.** Costs according to treatment stage: HER2 (+) neo/adjuvant or metastatic breast cancer – INEN.

Regime	Drugs	Course for one person with body surface area: 1.6	Total vials	Total price
**HER2 (+) neo/adjuvant breast cancer (estimated for 12 months)**				
TCH (six courses of trastuzumab – carboplatin – docetaxel)	Docetaxel	1 × 80 mg injectable + 1 × 20 mg injectable	6 × 80 mg injectable 12 × 20 mg injectable	S/. 811.50
	Carboplatin	1 × 450 mg injectable + 2 × 150 mg injectable	6 × 450 mg injectable 12 × 150 mg injectable	S/. 877.26
	IV Trastuzumab	2 injectable (loading dose) in the first course, then 1 injectable (maintenance dose)	18 × 440 mg injectable	S/. 51,502.50
	Total cost (2019): S/. 103,836.78, total cost (2020): S/. 53,191.26 **Cost reduction (%): 48.78%**			**S/. 53,191.26**
ACTH (AC four courses, 12 weekly paclitaxel doses – trastuzumab	Doxorubicin	2 × 50 mg injectable	8 × 50 mg injectable	S/. 152.00
	Cyclophosphamide	1 × 1,000 mg injectable	4 × 1,000 mg injectable	S/. 245.00
	Paclitaxel	1 × 100 mg injectable + 1 × 30 mg injectable	12 × 100 mg injectable 12 × 30 mg injectable	S/. 450.96
	IV Trastuzumab	Two injectable (loading dose) in the first course, then one injectable (maintenance dose)	18 × 440 mg injectable	S/. 51,502.50
	Total cost (2019): S/. 102,995.98, total cost (2020): S/. 52,350.46 **Cost reduction (%): 49.18%**			**S/. 52,350.46**
**HER2 (+) metastatic breast cancer (estimated for 6 months)**				
Trastuzumab – docetaxel	Docetaxel	1 × 80 mg injectable + 1 × 20 mg injectable	6 × 80 mg injectable 12 × 20 mg injectable	S/. 811.50
	IV Trastuzumab	Two injectable (loading dose) in the first course, then one injectable (maintenance dose)	7 × 440 mg injectable	S/. 20,028.75
				**S/. 20,840.25**
Trastuzumab – paclitaxel every 3 weeks (Q3W)	Paclitaxel	3 × 100 mg injectable	18 × 100 mg injectable	S/. 416.16
	IV Trastuzumab	Two injectable (loading dose) in the first course, then one injectable (maintenance dose)	7 × 440 mg injectable	S/. 20,028.75
				**S/. 20,444.91**

Number of vials estimated for a patient with an average weight of 60–70 kg. The IV trastuzumab dose (for all treatment stages: metastatic, adjuvant and neoadjuvant) is: 8 mg/kg (loading dose), followed by 6 mg/kg (maintenance dose) every 3 weeks

Source: Functional Insurance Unit. National Institute of Neoplastic Diseases (INEN)

^1^These amounts correspond to a value of 1 dollar ($1.00), equivalent to 3.50 sols (S/. 3.50)

ACTH: doxorubicin/cyclophosphamide/paclitaxel/trastuzumab

ACTH scheme: "AC for 4 courses, followed by weekly paclitaxel + trastuzumab"
